# Neighborhood Assets and Challenges for Black Older Adults With Dementia or Mild Cognitive Impairment

**DOI:** 10.1093/geroni/igaf122.2118

**Published:** 2025-12-31

**Authors:** Clara Scher

**Affiliations:** Weill Cornell Medicine, New York, New York, United States

## Abstract

Due to the detrimental impacts of structural racism, many Black older adults living with dementia or mild cognitive impairment in the United States are aging in communities with unequal access to essential resources. While previous research has examined how neighborhood environments impact those aging in community with dementia or mild cognitive impairment, few studies have specifically focused on experiences of older adults racialized as Black. Guided by the Black Family Socioecological Context Model, this study explored how neighborhood factors support or challenge the well-being of this population in the context of an urban city. Researchers recruited eight Black adults 65 and older with self-reported memory loss from social adult day centers across two New York City boroughs. Participants engaged in both a sit-down interview and a walking interview, which involved the participant guiding the researcher on a tour throughout their neighborhood. Reflexive thematic analysis and memoing were used to analyze interview transcripts and field notes. Findings emphasized how place-based community assets—such as social adult day centers, churches, and transportation services—enhanced participants’ well-being by providing access to essential resources, fostering emotional support, and strengthening social connections. Yet, community-level hazards—including crime and safety concerns, structural racism and ageism, and insufficient government investment—created barriers to accessing services and contributed to distress. Future participatory research should include larger, more geographically diverse samples to explore how community assets can be leveraged to enhance well-being and reduce the negative effects of neighborhood-based barriers among Black older adults with dementia or mild cognitive impairment.

